# MeCP2 interacts with the super elongation complex to regulate transcription

**DOI:** 10.1126/sciadv.adt5937

**Published:** 2025-11-26

**Authors:** Jun Young Sonn, Wonho Kim, Marta Iwanaszko, Yuki Aoi, Yan Li, Guantong Qi, Luke Parkitny, Janice L. Brissette, Lorin Weiner, Juan Botas, Ismael Al-Ramahi, Ali Shilatifard, Huda Y. Zoghbi

**Affiliations:** ^1^ Department of Molecular and Human Genetics, Baylor College of Medicine, Houston, TX, USA.; ^2^ Jan and Dan Duncan Neurological Research Institute, Texas Children’s Hospital, Houston, TX, USA.; ^3^ Howard Hughes Medical Institute, Baylor College of Medicine, Houston, TX, USA.; ^4^ Simpson Querrey Institute for Epigenetics and the Department of Biochemistry and Molecular Genetics, Northwestern University Feinberg School of Medicine, Chicago, IL, USA.; ^5^ Department of Cell Biology, State University of New York Downstate Health Sciences University, Brooklyn, NY, USA.; ^6^ Department of Molecular and Cellular Biology, Baylor College of Medicine, Houston, TX, USA.; ^7^ Center for Alzheimer’s and Neurodegenerative Diseases, Baylor College of Medicine, Houston, TX, USA.; ^8^ Department of Pediatrics, Baylor College of Medicine, Houston, TX, USA.; ^9^ Department of Neuroscience, Baylor College of Medicine, Houston, TX, USA.; ^10^ Department of Neurology, Baylor College of Medicine, Houston, TX, USA.

## Abstract

Loss-of-function mutations in methyl-CpG binding protein 2 (*MECP2*) cause Rett syndrome. While we know that MeCP2 binds to methylated cytosines on DNA, the full breadth of the molecular mechanisms by which MeCP2 regulates gene expression remains incompletely understood. Here, using a genetic modifier screen, we identify the super elongation complex, a P-TEFb–containing elongation factor that releases promoter-proximally paused RNA polymerase II, as a genetic interactor of *MECP2*. MeCP2 physically interacts with SEC subunits and directly binds AFF4, the scaffold of the SEC, via the transcriptional repression domain. Furthermore, MeCP2 facilitates the binding of AFF4 on a subset of genes in the mouse brain regulating synaptic plasticity and concordantly promotes the binding of RNA polymerase II on these genes. Last, while haploinsufficiency of *Aff4* does not exhibit any behavioral deficits in mice, it exacerbates the impaired contextual learning behavior of *Mecp2* hypomorphic mice. We propose a previously unknown mechanism by which MePC2 regulates gene expression underlying synaptic plasticity.

## Teaser

MeCP2 interacts with the super elongation complex, a transcriptional activator, to regulate the expression of synaptic genes.

## INTRODUCTION

Rett syndrome (RTT) is a devastating neurodevelopmental disorder resulting from de novo loss-of-function mutations in the X-linked gene, *MECP2* (*1*). Typically, girls with classical RTT develop normally for the first 12 to 18 months of life but then lose their acquired language and motor skills and gradually develop stereotypies, motor difficulties, autism, seizures, and autonomic dysfunction (*2*, *3*).

The MeCP2 protein is present in a variety of tissues in mammals, with the highest levels in neurons of the central nervous system (*4*, *5*). MeCP2 levels are initially low during embryonic development but increase dramatically after birth and are maintained at a high level throughout adulthood (*6*, *7*). The period of this increase in MeCP2 levels coincides with the critical stage of postnatal brain maturation during which many synapses form and experiences refine synaptic connections (*8*). Therefore, MeCP2 is believed to be critical for the function of mature neurons. Mouse models of RTT demonstrate smaller brain size without detectable cell loss, reduction in number and length of dendrites, and a reduction in dendritic spine numbers (*9*, *10*). These lines of evidence demonstrate the importance of MeCP2 in the proper regulation of synaptic integrity and function.

At the molecular level, a consensus of MeCP2 function and how its dysregulation leads to disease is currently lacking. MeCP2 contains five protein domains: N-terminal domain (NTD), methyl-CpG–binding domain (MBD), intervening domain (ID), transcriptional repression domain (TRD), and the C-terminal domain (CTD). Through the MBD, MeCP2 binds to methylated cytosines on genomic DNA (*11*–*16*) and recruits corepressors such as the Sin3A and NCoR complexes through its TRD (*17*, *18*). In addition, an early study has shown that the TRD of MeCP2 represses transcription in vitro (*19*). On the basis of these results, MeCP2 has traditionally been viewed as a repressor of transcription. However, several subsequent studies have suggested that MeCP2 may also play a role in activation of gene expression. First, transcriptomic studies in the hypothalamus of *Mecp2* null mice have shown that most of the differentially expressed genes are down-regulated while some are up-regulated, and those same genes are inversely altered in the overexpression mouse model (*20*). This bidirectional inverse gene expression changes in mouse models of loss and gain of MeCP2 have been reproduced in follow-up studies (*21*, *22*). Second, our laboratory has previously found that TCF20, which is associated with transcriptional activation, interacts with MeCP2 (*23*). Last, MeCP2 has recently been shown to bind to hypomethylated promoter-proximal regions and globally recruits RNA polymerase II (RNA pol II) (*24*).

We rationalized that using a genetic approach to identify the repertoire of functional interactors of MeCP2 would enable us to gain a deeper understanding of how MeCP2 regulates gene expression. *Drosophila* lacks an orthologous gene to *MECP2* and shows minute levels of methylation on their genomic DNA (*25*), yet the ectopic overexpression of human *MECP2* (h*MECP2*) in *Drosophila* can recapitulate the key functional characteristics of the protein. For example, overexpressed h*MECP2* associates with *Drosophila* chromatin, genetically interacts with the *Drosophila* orthologs of known interacting partners including subunits of the Sin3A, NCoR, and SWI/SNF complexes, and can be phosphorylated at Ser^423^ as in mammals (*26*). These lines of evidence suggest that the biology of the MeCP2 interactome can be interrogated in *Drosophila*, giving us a unique opportunity to use this genetic tool to find genetic interactors of *MECP2*.

Overexpressing h*MECP2* in the *Drosophila* eye caused a rough-eye phenotype, allowing us to screen for genes whose knockdown can either suppress or aggravate this phenotype. We screened for chromatin-associated genes that were conserved in *Drosophila* and validated subunits of previously identified interactor complexes, including the NCoR complex, as genetic interactors of h*MECP2*. Notably, we identified subunits of the SEC as previously unknown genetic interactors of h*MECP2*. The SEC is a transcriptional activator complex composed of AFF1/AFF4, AF9/ENL, ELL1/ELL2, EAF1/EAF2, and P-TEFb that phosphorylates Ser^2^ of the CTD of promoter-proximally paused RNA pol II (*27*–*29*). Upon stimuli such as heat shock or developmental cues, the SEC releases RNA pol II into the gene body. We find that MeCP2 interacts with SEC subunits both in vitro and in vivo and directly binds AFF4 (the core scaffold of the SEC) via the TRD domain. Furthermore, we find that MeCP2 facilitates the recruitment of AFF4 on a subset of highly expressed genes in the mouse cortex associated with regulation of synaptic function and plasticity and a concordant depletion of RNA pol II binding on the gene body of these genes in the *Mecp2* null mouse cortex. Last, we find that *Mecp2* and *Aff4* genetically interact in mice to specifically regulate contextual learning and memory behavior. These data collectively suggest the functional interaction between MeCP2 and the SEC in the brain to regulate transcription supporting healthy neurological function.

## RESULTS

### Knockdown of *Drosophila* SEC subunits suppress the h*MECP2*-induced rough-eye phenotype

**Fig. 1.**
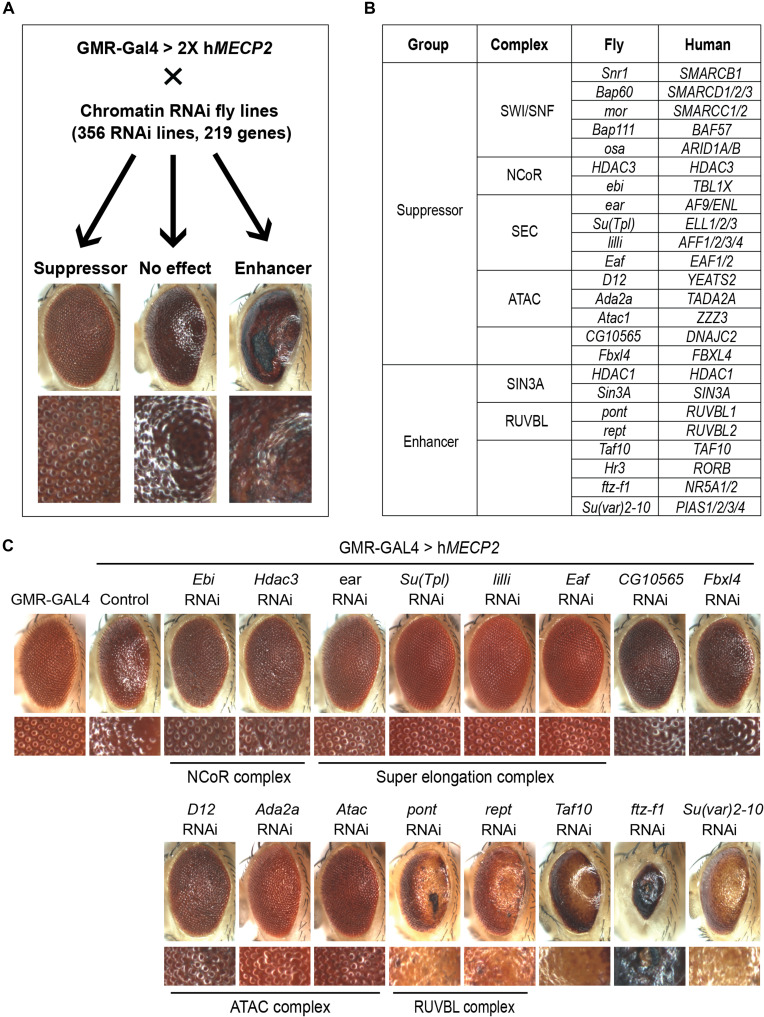
*MECP2* genetic modifier screen in *Drosophila*. (**A**) Schematic to find genetic modifiers of h*MECP2* in *Drosophila*. Images in bottom row represent magnifications of the respective images on the top. Eye images under “No effect” represent a control fly overexpressing two copies of h*MECP2* together with *Luciferase* RNAi. Eye images under “Suppressor” represent a fly in which knockdown of *ebi* (*Drosophila* ortholog of *TBL1X* and subunit of the NCoR complex) mitigates the h*MECP2*-induced rough-eye phenotype. Eye images under “Enhancer” represent a fly in which knockdown of *HDAC1* (subunit of the Sin3A complex) aggravates the h*MECP2*-induced rough-eye phenotype. (**B**) Table showing *Drosophila* candidate modifier genes and their human orthologues, knockdown of which either mitigates (suppressor) or aggravates (enhancer) the h*MECP2*-induced rough eye phenotype. (**C**) Light microscope images of eyes with no overexpression (GMR-GAL4) and overexpression of h*MECP2* (control) at 28.5°C. RNAi-mediated knockdown of NCoR complex subunits, SEC subunits, *CG10565*, *Fbxl4*, and ATAC complex subunits mitigate, while knockdown of RUVBL complex subunits, *Taf10*, *ftz-f1*, and *Su(var)2-10* aggravate the rough-eye phenotype. Image of *Hr3* knockdown is not shown as it caused lethality.

To find chromatin-associated genes whose knockdown could modify the h*MECP2*-induced rough eye, we screened a total of 356 RNA interference (RNAi) lines that target 219 genes in *Drosophila* (Fig. 1A and table S1). From this screen, we identified 16 genes that mitigated (suppressor) and 8 genes that aggravated (enhancer) this phenotype (Fig. 1B). Consistent with previous results (*26*), we found that knockdown of previously known interactors modified the h*MECP2*-induced rough-eye phenotype. For example, knockdown of subunits of the NCoR and SWI/SNF complexes mitigated, while knockdown of subunits of the Sin3A complex aggravated, the h*MECP2*-induced rough-eye phenotype (Fig. 1C and fig. S1A). Besides the known interactors, we also identified subunits of the SEC [*ear*, *Su(Tpl)*, *lilli*, and *Eaf*], *CG10565*, *Fbxl4*, Ada2a-containing (ATAC) complex (*D12*, *Ada2a*, and *Atac1*), RUVBL complex (*pont* and *rept*), *Taf10*, *ftz-f1*, and *Su(var)2-10* as previously unknown interactors of h*MECP2* (Fig. 1C). Among the interactors, the SEC caught our attention for two reasons. First, quantification of the rough-eye phenotypes revealed that knockdown of SEC subunits most strongly suppressed the h*MECP2*-induced rough-eye phenotype (fig. S1B), suggesting a strong genetic interaction between *MECP2* and the SEC. Second, since the SEC facilitates transcription, we hypothesized that exploring the interaction between MeCP2 and the SEC could reveal a previously unknown mechanism underlying MeCP2-mediated transcriptional regulation. For these reasons, we decided to focus on the interaction between MeCP2 and the SEC going forward.

Before proceeding, we sought to determine whether knockdown of subunits of the *Drosophila* SEC could affect the GAL4-UAS system and potentially result in an artificial rescue phenotype. In flies expressing green fluorescent protein (GFP) under the GMR-GAL4 driver, we knocked down SEC subunits via RNAi and measured the GFP protein levels in the head using Western blot. Knockdown of *Drosophila* SEC subunits using multiple RNAis resulted in the following GFP levels compared to the control (VK37): *lilli* = 17 to 45%; *Su(Tpl)* = 66 to 78%; *ear* = 93 to 120%; and *Eaf* = 98%, while suppressing the h*MECP2*-induced rough-eye phenotype (fig. S1, C and D). To exclude the possibility that knockdown of *lilli* suppressed the rough-eye phenotype due to a down-regulation of GAL4-UAS activity, we tested the effect of a third RNAi line against *lilli* (*lilli* RNAi #3) on the h*MECP2*-induced rough-eye phenotype and GAL4-UAS activity. We observed that *lilli* RNAi #3 decreased GFP levels by only ~25% while strongly suppressing the rough-eye phenotype (fig. S1, E and F). Collectively, these results suggest that an alteration in the genetic interaction between *MECP2* and the SEC is responsible for suppressing the h*MECP2*-induced rough-eye phenotype.

### MeCP2 physically interacts with the SEC and RNA pol II

To determine whether MeCP2 can physically associate with the SEC, we coexpressed C-terminal yellow fluorescent protein (YFP)–tagged MeCP2 (MeCP2-cYFP) with N-terminal YFP–tagged SEC subunits (AFF4-nYFP, ENL-nYFP, and EAF1-nYFP) in human embryonic kidney (HEK) 293T cells and performed bimolecular fluorescence complementation (BiFC) (*30*). Coexpression of MeCP2-cYFP with Ppp6c-nYFP (negative control), a protein that is not known to interact with MeCP2, did not produce YFP-positive cells when analyzed by fluorescence-activated cell sorting (FACS) (fig. S2A). However, coexpression of MeCP2-cYFP with TBL1X-nYFP (positive control), a subunit of the NCoR complex known to directly bind to MeCP2 (*31*), resulted in YFP-positive cells, thereby demonstrating the specificity of our assay. When MeCP2-cYFP was coexpressed with nYFP-tagged SEC subunits, YFP-positive cells were detected, suggesting a physical interaction between MeCP2 and the SEC in cells. Confocal microscopy images of the YFP signal further confirmed that MeCP2 interacts with the SEC subunits within the nucleus, as expected (fig. S2B).

**Fig. 2.**
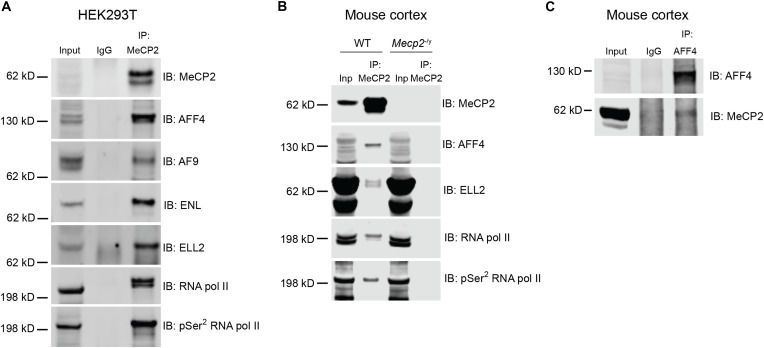
MeCP2 physically interacts with the SEC. (**A**) Endogenous MeCP2 interacts with endogenous SEC subunits (AFF4, AF9, ENL, and ELL2) and RNA pol II in HEK293T cells. Normal mouse immunoglobulin G was used as a negative control. (**B**) Endogenous MeCP2 interacts with SEC subunits (AFF4 and ELL2) and RNA pol II in the cortex of WT mouse at 7 weeks of age. (**C**) Reverse IP of endogenous AFF4 from WT cortical lysate and immunoblotting against MeCP2. Different brightness settings were used for the top and bottom blots because of the relatively weaker MeCP2 co-IP band intensity compared to the AFF4 IP band intensity. Immunoblotting against AFF4 for (A) and (B) was performed with the Bethyl Laboratories antibody (A302-538A), whereas IP and immunoblotting for AFF4 for (C) was performed with the Proteintech antibody (14662-1-AP).

Next, to determine whether MeCP2 and the SEC interact under physiological contexts, we immunoprecipitated (IPed) endogenous MeCP2 from HEK293T cell lysates and then probed for the coimmunoprecipitation (co-IP) of endogenous SEC subunits. We observed that AFF4, AF9, ENL (AF9 paralog), and ELL2 co-IPed with MeCP2 (Fig. 2A and fig. S2C). Since the SEC phosphorylates RNA pol II for productive elongation (*27*–*29*), we rationalized that MeCP2 might also associate with RNA pol II. We observed that both total RNA pol II and its elongating form (pSer^2^ RNA pol II) co-IPed with MeCP2.

We next sought to determine whether MeCP2 interacts with the SEC and RNA pol II in vivo. To this end, we IPed MeCP2 from cortical lysates of wild-type (WT) and *Mecp2* null mice. Before probing for the co-IP of SEC subunits and RNA pol II, we first validated our IP condition by probing for TBL1X (positive control) and vinculin (negative control), a protein that is known to be localized exclusively in the cytoplasm and therefore would not interact with MeCP2. We observed that MeCP2 co-IPed with TBL1X, but not vinculin (fig. S2D), thereby confirming that our IP condition accurately depicts MeCP2 interactors in vivo. Under this condition, we observed that MeCP2 co-IPed with SEC subunits (AFF4 and ELL2) and both total and pSer^2^ RNA pol II (Fig. 2B and fig. S2E). To further validate the interaction between MeCP2 and the SEC in vivo, we reversely IPed for AFF4 and probed for the co-IP of MeCP2. Although the co-IP band was faint, it was reproducible (Fig. 2C and fig. S2F). Together, these biochemical data indicate a physical interaction between MeCP2 and the SEC.

Besides the SEC, there are two additional SEC-like complexes, SEC-like 2 (SEC-L2) and SEC-like 3, which use AFF2 and AFF3 paralogs as their scaffolds, respectively (*32*). To determine whether MeCP2 also interacts with SEC-L2 and SEC-L3, we IPed MeCP2 from cortical lysates of WT mice and probed for the co-IP of AFF2 and AFF3. However, we did not observe an interaction between these paralogs and MeCP2 (fig. S2G), suggesting that MeCP2 preferentially interacts with the AFF4-associated SEC.

### The TRD is essential for MeCP2’s direct binding to AFF4

**Fig. 3.**
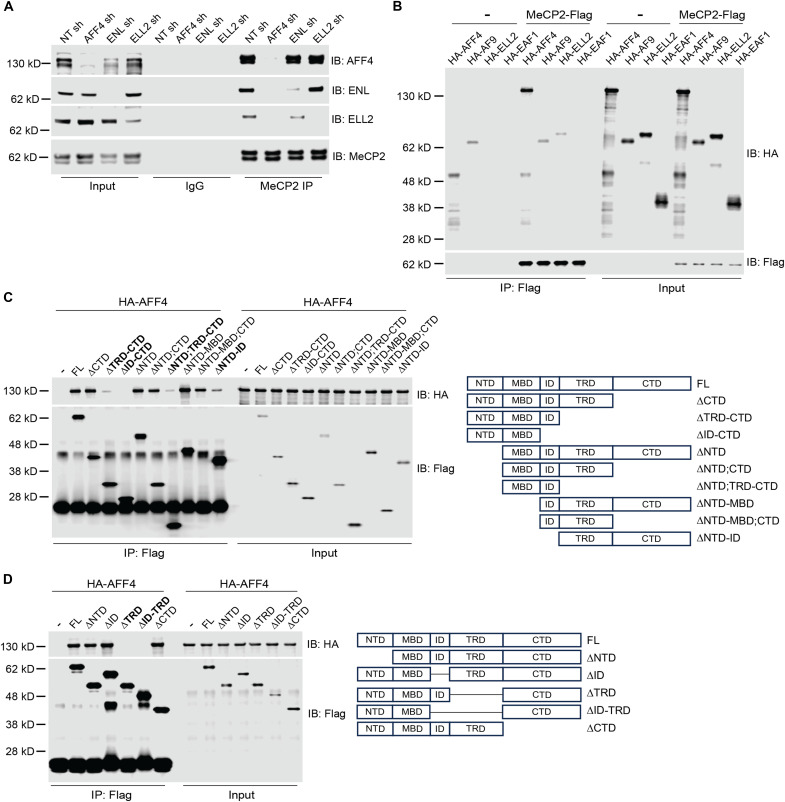
The TRD is required for MeCP2’s direct binding to AFF4. (**A**) Short hairpin–mediated knockdown of *AFF4* in HEK293T cells abolishes the interaction between MeCP2 and the remaining subunits of the SEC (ENL and ELL2). While knockdown of *ENL* mildly dissociates MeCP2’s interaction with ELL2, it does not affect its interaction with AFF4. Knockdown of *ELL2* does not affect the interaction between MeCP2 and the remaining subunits of the SEC. NT sh is a nontargeting short-hairpin negative control. (**B**) In vitro binding assay between recombinant FLAG-tagged MeCP2 and HA-tagged SEC subunits. (**C**) In vitro binding assay between FLAG-tagged MeCP2 fragments and HA-tagged AFF4. The fragments that show a decrease in interaction with HA-AFF4 are highlighted in bold. Diagram on the right shows a map of the MeCP2 fragments tested. NTD, N-terminal domain; MBD, methyl-CpG–binding domain; ID, intervening domain; TRD, transcriptional repression domain; CTD, C-terminal domain. (**D**) In vitro binding assay between AFF4 and MeCP2 with deletions in the NTD, ID, TRD, ID and TRD, and CTD. The domains critical for interaction are highlighted in bold.

To determine the SEC subunit that mediates the interaction with MeCP2, we genetically knocked down three different subunits of the SEC (*AFF4*, *ENL*, and *ELL2*) in HEK293T cells using short hairpin RNAs and tested for the interaction between MeCP2 and the rest of the SEC subunits. Knockdown of *AFF4* substantially reduced the co-IP of other SEC subunits (ENL and ELL2) and MeCP2 (Fig. 3A). In contrast, knocking down *ENL* did not affect the co-IP of AFF4 while mildly reducing the co-IP of ELL2. Furthermore, knocking down *ELL2* did not affect MeCP2’s interaction with other SEC subunits.

In parallel, we also examined the direct binding between MeCP2 and SEC subunits in vitro. Using an in vitro–coupled transcription and translation (in vitro TnT) system, we generated FLAG-tagged MeCP2 (MeCP2-FLAG) and hemagglutinin (HA)–tagged SEC subunits (HA-AFF4, HA-AF9, HA-ELL2, and HA-EAF1). Subsequently, MeCP2-FLAG was incubated individually with each of the recombinant SEC subunits followed by IP using FLAG antibody–conjugated beads. We observed that HA-AFF4 came down only in the presence of MeCP2-FLAG (Fig. 3B). Further, we observed that HA-AF9 came down even in the absence of MeCP2-FLAG, indicating that FLAG-conjugated beads can nonspecifically pull down AF9. However, in the presence of MeCP2-FLAG, HA-AF9 did not come down more, suggesting that MeCP2 does not bind to AF9. We noticed that a small amount of HA-ELL2 came down with MeCP2-FLAG, suggesting that MeCP2 weakly interacts with ELL2. Last, HA-EAF1 did not come down either in the absence or presence of MeCP2-FLAG, indicating that EAF1 does not interact with MeCP2. Collectively, these results suggest that MeCP2 preferentially and directly binds to AFF4.

Next, to identify the protein domain within MeCP2 that is required for its interaction with AFF4, we generated FLAG-tagged MeCP2 fragments with various domain deletions using the in vitro TnT system and incubated the recombinant fragments individually with HA-AFF4. We found that while deleting the NTD, CTD, and MBD did not affect MeCP2’s interaction with AFF4, deleting the TRD greatly reduced MeCP2’s binding to AFF4 (Fig. 3C). In addition, we noticed that deletion of the ID with other domains mildly affected the binding. However, deleting only the ID did not affect MeCP2’s binding to AFF4, while deleting only the TRD greatly reduced the binding (Fig. 3D). While such manipulation could affect the overall conformation of MeCP2 and thereby affect its binding to AFF4, it nevertheless indicates that the TRD of MeCP2 is essential for its interaction with AFF4.

### MeCP2 facilitates the binding of AFF4 on a subset of genes while promoting the global binding of RNA pol II

Given that MeCP2 physically interacts with the SEC, we hypothesized that MeCP2 recruits the SEC to facilitate RNA pol II binding. To test this hypothesis, we decided to assess whether the loss of MeCP2 can affect the genome-wide binding of AFF4 and RNA pol II in the mouse brain using chromatin IP sequencing (ChIP-seq). Before proceeding, we first tested whether our antibody could specifically detect AFF4 in the mouse brain. Since AFF4 binding is induced on genes that respond to stimuli (*27*–*29*), we decided to evaluate the binding of AFF4 in the mouse brain under baseline and activated conditions. To this end, we injected either saline or kainic acid (KA) into *Camk2a*-Cre (Cre control) and *Camk2a*-Cre > *Aff4*^fl/fl^ (*Aff4* cKO) mice and performed AFF4 ChIP-seq on the cortical lysates. We observed that a short treatment (45 min) of KA in the Cre control mouse induced binding of AFF4 on a small cluster of genes (*n* = 75), while decreasing binding on a separate cluster of genes (*n* = 370; fig. S3A and table S2). KA injection in the *Aff4* cKO mouse suppressed the induction of AFF4 binding on select genes, most probably due to the excitatory neuron–specific activity of *Camk2a*-Cre. This is exemplified by the observation that conditional knockout of *Aff4* abolished the KA-induced binding of AFF4 on *Egr3*, a gene that is heavily expressed in excitatory neurons, but not on other genes that are expressed mainly in other cell types such as inhibitory neurons, pericytes, and endothelial cells (fig. S3, B and C). Nevertheless, the fact that we see a substantial suppression of the KA-induced binding of AFF4 in the *Aff4* cKO mouse demonstrates the validity of our antibody.

**Fig. 4.**
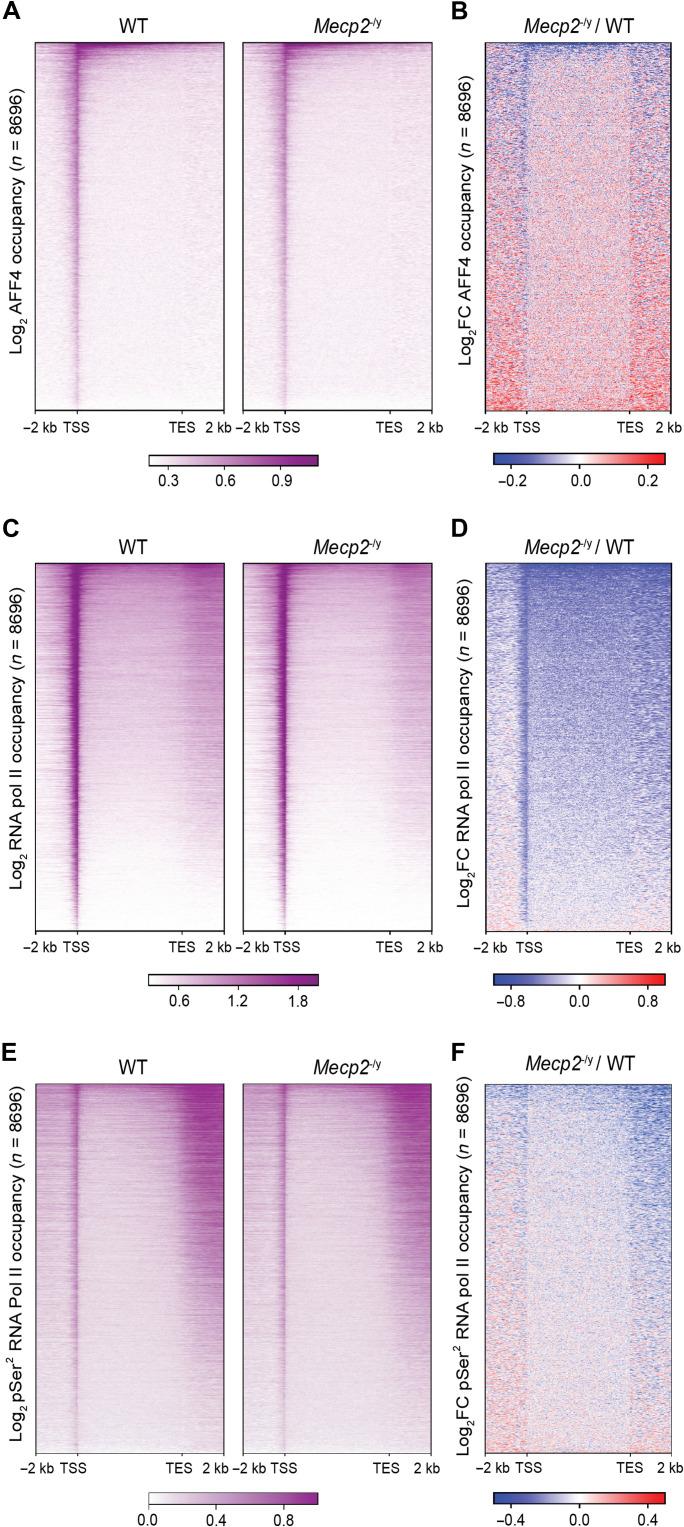
MeCP2 facilitates the binding of AFF4 on a subset of genes while globally promoting the binding of RNA pol II. (**A**) Global heatmap of log_2_-transformed occupancy of AFF4 in the cortex of WT and *Mecp2* null mice. (**B**) Global heatmap of log_2_ fold change of AFF4 occupancy in *Mecp2* null mouse compared to WT mouse. (**C**) Global heatmap of log_2_-transformed occupancy of RNA pol II in the cortex of WT and *Mecp2* null mice. (**D**) Global heatmap of log_2_ fold change of RNA pol II occupancy in *Mecp2* null mouse compared to WT mouse. (**E**) Global heatmap of log_2_-transformed occupancy of pSer^2^ RNA pol II in the cortex of WT and *Mecp2* null mice. (**F**) Global heatmap of log_2_ fold change of pSer^2^ RNA pol II occupancy in *Mecp2* null mouse compared to WT mouse. *n* = 8696 RNA pol II–bound genes are represented in all heatmaps.

Comparing the genome-wide binding of AFF4 in the cortex of WT and *Mecp2* null mice, we observed a reduction in binding of AFF4 on a subset of genes in all three biological replicates (Fig. 4, A and B, and fig. S4A). To ensure that this reduction in binding was not due to a decrease in protein levels of AFF4, we measured AFF4 levels in the cortex of WT and *Mecp2* null mice via Western blot. We observed that AFF4 protein levels were comparable between WT and *Mecp2* null mice (fig. S4B). In addition, when we compared the genome-wide binding of RNA pol II, unexpectedly, we found a global reduction in *Mecp2* null mice in three biological replicates (Fig. 4, C and D, and fig. S4C). To confirm this observation, we also compared the genome-wide binding of pSer^2^ RNA pol II and found a similar global reduction in *Mecp2* null mice in three biological replicates (Fig. 4, E and F, and fig. S4D). These results collectively suggest that MeCP2 globally supports the binding of RNA pol II while the interaction between MeCP2 and the SEC supports the binding of RNA pol II on a subset of genes.

### MeCP2 supports the expression of genes involved in synaptic processes via the SEC

**Fig. 5.**
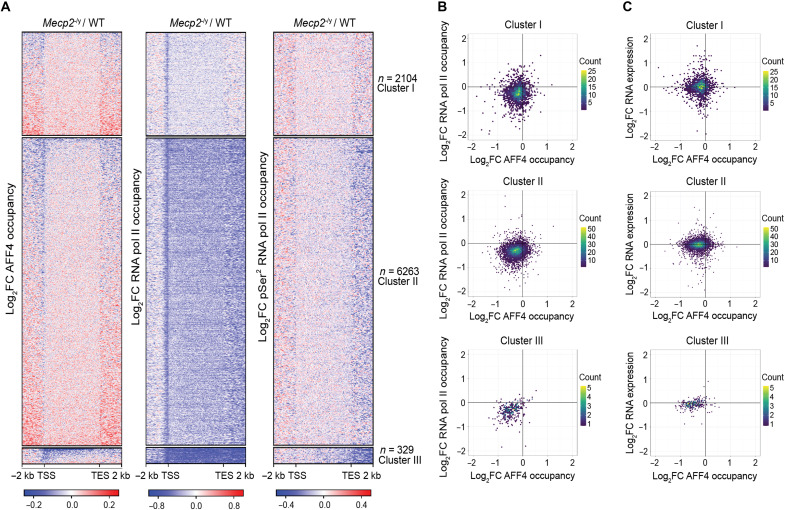
MeCP2 supports the expression of SEC-regulated genes. (**A**) Global heatmap showing the log_2_ fold change of AFF4, RNA pol II, and pSer^2^ RNA pol II binding in the *Mecp2* null cortex based on hierarchical clustering. (**B**) Two-dimensional plots for clusters in (A), showing the correlation between AFF4 and RNA pol II binding as the median of *Mecp2* null versus WT ratios across matched animal pairs. *n* = 3 biological replicates. Color scale indicates the gene count. Spearman’s correlation values: ρ = 0.081; *P* = 0.00019 (cluster I), ρ = 0.13; *P* < 2.2 × 10^−16^ (cluster II), ρ = 0.34; *P* = 4.8 × 10^−10^ (cluster III). (**C**) Two-dimensional plots for clusters in (A), showing the correlation between AFF4 binding and RNA expression as the median of *Mecp2* null versus WT ratios across matched animal pairs. Color scale indicates the gene count. *n* = 3 biological replicates for AFF4 ChIP-seq and *n* = 6 biological replicates for RNA-seq. Spearman’s correlation values: cluster I ρ = 0.026, *P* = 0.23; cluster II ρ = 0.063, *P* = 7.9 × 10^−07^; cluster III ρ = 0.27, *P* = 5.3e-07.

To dissect the interplay between MeCP2, SEC, and RNA pol II, we clustered the genes based on the change of AFF4 and RNA pol II binding in *Mecp2* null mice. This generated three independent clusters: clusters I, II, and III (Fig. 5A, fig. S5A, and table S3). Cluster I (*n* = 2104 genes) displayed a mixture of genes that exhibited both increased and decreased binding of AFF4 upstream of the transcription start site (TSS) and downstream of the transcription end site (TES). For total RNA pol II, genes in this cluster showed a decrease in binding on the TSS while pSer^2^ RNA pol II did not show a change in binding. Cluster II (6263 genes) displayed a similar mixture of genes with both increased and decreased binding of AFF4 upstream of the TSS and downstream of the TES. However, total RNA pol II binding was decreased not only on the TSS but also across the gene body and beyond the TES, while pSer^2^ RNA pol II binding was decreased mostly downstream of the TES. Last, although cluster III was composed of only 329 genes, AFF4 binding was decreased on the TSS and across the gene body. Concordantly, this cluster of genes showed the greatest reduction in total RNA pol II and pSer^2^ RNA pol II binding across the gene body and downstream of the TES. To determine whether the change in AFF4 and RNA pol II binding in *Mecp2* null animals were correlated to each other, we plotted the gene body change in AFF4 binding versus RNA pol II binding across the three clusters. While Spearman’s correlation analysis did not reveal a meaningful correlation for clusters I and II (cluster I: ρ = 0.081, *P* = 0.00019 and cluster II: ρ = 0.13, *P* = 2.2 × 10^−16^), it revealed a moderate correlation for cluster III (cluster III: ρ = 0.34, *P* = 4.8 × 10^−10^; Fig. 5B).

To determine whether the change in binding of AFF4 correlates with change in gene expression, we also performed RNA sequencing (RNA-seq) on the cortex of WT and *Mecp2* null mice. Principal components analysis revealed that the WT samples segregated from the *Mecp2* null samples (fig. S5B), demonstrating the validity of our RNA-seq results. When we plotted the change in AFF4 binding versus RNA expression in *Mecp2* null animals, we again did not observe a meaningful correlation for clusters I and II (cluster I: ρ = 0.026, *P* = 0.23 and cluster II: ρ = 0.063, *P* = 7.9 × 10^−07^) but observed a moderate correlation for cluster III (cluster III: ρ = 0.27, *P* = 5.3 × 10^−07^; Fig. 5C). Furthermore, when we calculated the average expression of genes in each cluster, we found that cluster I had a slight increase while clusters II and III were decreased in *Mecp2* null mice compared to WT mice, with cluster III showing the greatest magnitude of change (fig. S5C). Collectively, these evidence suggest the possibility that the decreased binding of AFF4 contributes to the decreased gene body binding of RNA pol II and expression of cluster III genes.

Gene ontology analysis revealed that cluster I was enriched for genes involved in vascular process in circulatory system, extracellular structure organization, sensory organ morphogenesis, and adaptive immune response (fig. S5D). Cluster II was enriched for genes involved in various neuron developmental processes such as dendrite development, axonogenesis, regulation of neurogenesis, and forebrain development. Last, cluster III was enriched for genes involved in synaptic processes such as regulation of synaptic plasticity, signal release from synapse, neurotransmitter transport, and cognition. Examples of genes in this cluster included *Homer1*, *Jun*, and *Sik1* (fig. S5E).

Our analysis of the average binding levels of AFF4, RNA pol II, and pSer^2^ RNA pol II across the clusters revealed that cluster III had the highest level of binding in WT mice (fig. S5F). Moreover, cluster III also showed the highest level of RNA expression (fig. S5G). Together, these data suggest that the interaction between MeCP2 and the SEC supports the transcription of the most highly expressed genes involved in the regulation of synaptic function and plasticity in the mouse brain.

### *Aff4* haploinsufficiency exacerbates the impaired contextual learning and memory behavior of *Mecp2* hypomorphic mice

**Fig. 6.**
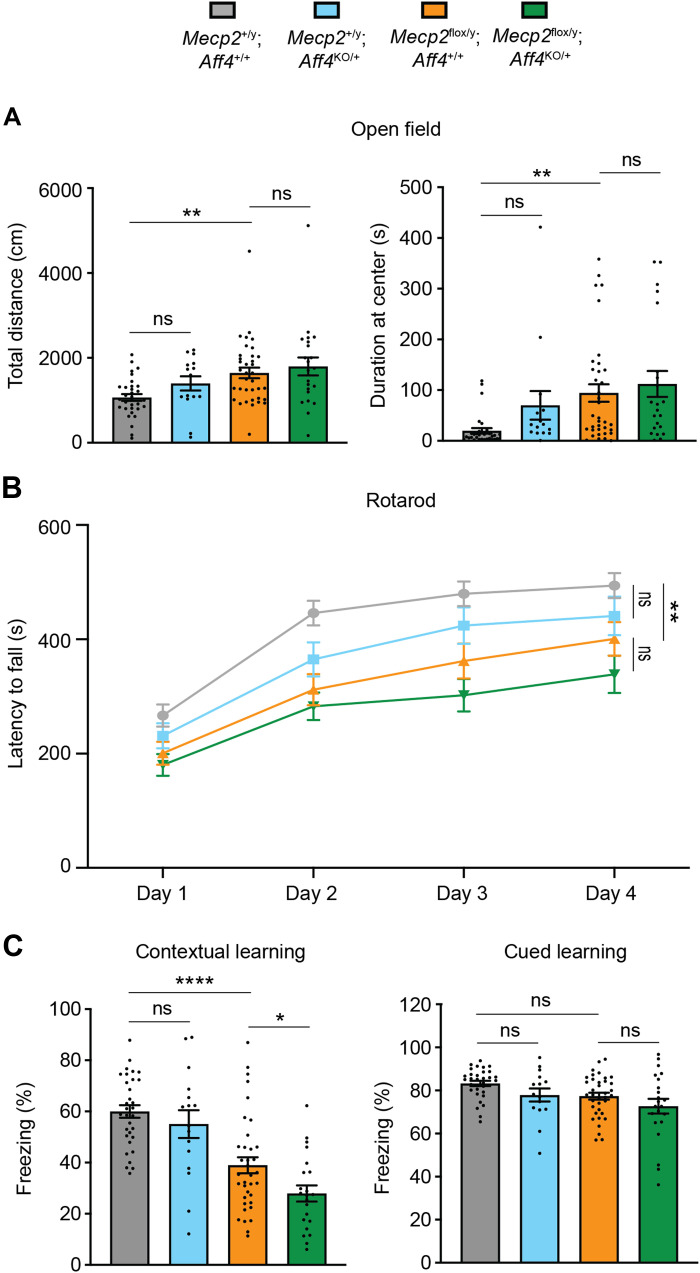
*Mecp2* and *Aff4* genetically interact to regulate learning and memory behavior in mice. (**A**) Open-field assessment of activity levels and duration at the center of the arena beginning at 10 weeks of age. (**B**) Rotarod assay measures motor-coordination ability. (**C**) Fear conditioning assay measuring learning and memory capabilities in both contextual and cued settings. For open-field and fear conditioning assays [(A) and (C)], *n* = 32 WT mice (gray); *n* = 16 *Aff4* heterozygous knockout mice (blue); *n* = 36 *Mecp2* hypomorphic mice (orange); *n* = 22 double-mutant mice (green). For rotarod (B), *n* = 22 WT mice; *n* = 14 *Aff4* heterozygous knockout mice; *n* = 29 *Mecp2* hypomorphic mice; *n* = 19 double-mutant mice. ns, not significant; *P < 0.05, **P < 0.01, ****P < 0.0001.

Given that MeCP2 facilitates the recruitment of AFF4 to regulate the expression of genes involved in synaptic processes, we hypothesized that the two might functionally interact to regulate neurological function. To test this hypothesis, we subjected male WT mice (*Mecp2*^+/y^; *Aff4*^+/+^), *Aff4* haploinsufficient mice (*Mecp2*^+/y^; *Aff4*^KO/+^), and *Mecp2* hypomorphic mice (*Mecp2*^fl/y^; *Aff4*^+/+^), which have 50% MeCP2 levels (*33*), and double-mutant (*Mecp2*^fl/y^; *Aff4*^KO/+^) mice to several behavioral tests between 10 and 12 weeks of age. We first measured the body weight and found that *Aff4* haploinsufficient mice had significantly increased body weight compared to WT mice (fig. S6). Similarly, *Mecp2* hypomorphic mice also had significantly increased body weight compared to WT mice; however, the body weight of the double-mutant mice was comparable to those of *Mecp2* hypomorphic mice. On the open-field assay, *Aff4* haploinsufficient mice demonstrated similar activity levels and duration at the center compared to WT mice, whereas *Mecp2* hypomorphic mice were hyperactive and stayed longer at the center of the arena (Fig. 6A). The double-mutant mice showed comparable activity levels and duration at the center to *Mecp2* hypomorphic mice. It was a similar scenario on the rotarod, where *Aff4* haploinsufficient mice exhibited similar motor coordination performance compared to WT mice, whereas *Mecp2* hypomorphic mice exhibited decreased performance (Fig. 6B). Again, the double-mutant mice exhibited similar motor coordination performance compared to *Mecp2* hypomorphic mice. Last, on the fear conditioning test, we observed that *Aff4* haploinsufficient mice exhibited similar contextual learning and memory capabilities compared to WT mice, whereas *Mecp2* hypomorphic mice exhibited a significant impairment (Fig. 6C). Interestingly, the double-mutant mice exhibited a significant decrease in contextual learning and memory performance compared to *Mecp2* hypomorphic mice. For cued learning and memory, we observed that neither *Aff4* haploinsufficient nor *Mecp2* hypomorphic mice exhibited impairments compared to WT mice, and the double-mutant mice did not show a further impairment compared to *Mecp2* hypomorphic mice. These data collectively suggest that MeCP2 and AFF4 functionally interact to regulate contextual learning and memory behavior in mice.

## DISCUSSION

Through a genetic modifier screen in *Drosophila*, we have not only confirmed previously known interactors of MeCP2 such as subunits of the Sin3A, NCoR, and SWI/SNF complexes as genetic interactors but also found several previously unknown partners. This underscores the reliability and power of our genetic screening approach to interrogate the molecular function of MeCP2. From this screen, we identified the SEC, a bona fide transcription elongation factor, as a previously unknown interactor of MeCP2. Having identified the transcription factor SEC as an MeCP2 interactor adds a layer of mechanism by which MeCP2 regulates gene expression. We find that on the genes that MeCP2 facilitates the recruitment of the SEC to, the loss of MeCP2 causes the greatest decrease in RNA pol II binding in their gene body. This points toward the possibility that the interaction between MeCP2 and the SEC may be contributing to the release of promoter-proximally paused RNA pol II into the gene body for productive elongation. It is also interesting that the cluster of genes (cluster III) regulated by the interaction between MeCP2 and the SEC includes genes involved in the modulation of activity-dependent synaptic plasticity such as *Homer1*, *Sik1*, and *Jun*. The expression of these genes is known to be induced by neuronal activity, which in turn contributes to the modulation of synaptic plasticity (*34*–*38*). We observed that KA-induced activation of the mouse brain caused increased binding of AFF4 on these genes. This suggests that the SEC is a critical factor in the brain coordinating the release of RNA pol II in response to neuronal activity to modulate synaptic plasticity. This is especially convincing given our observation that MeCP2 and AFF4 functionally interact in mice to support contextual learning and memory behavior, a process that is heavily dependent on activity-dependent synaptic plasticity.

There have been many questions in the field regarding how MeCP2 regulates transcription. Previously, MeCP2 has been proposed to function as a roadblock on the gene body, thereby impeding the elongation of RNA pol II (*39*, *40*). However, the study by Boxer *et al.* (*41*), which measured the rate of RNA pol II elongation in the forebrain of WT and *Mecp2* null mice, showed that loss of MeCP2 does not affect the elongation rate of RNA pol II. Instead, they observed that loss of MeCP2 leads to increased RNA pol II binding at the TSS of highly methylated long genes, suggesting that MeCP2 represses transcriptional initiation. Recently, Yi *et al.* reported that MeCP2 occupies hypomethylated CpG-rich promoter-proximal regions and globally recruits RNA pol II in human embryonic stem cell–derived neurons (*24*). Our data provide in vivo evidence that MeCP2 globally supports the binding of RNA pol II. Of the 8696 RNA pol II–bound genes that were analyzed, approximately 90% of them exhibited decreased binding of RNA pol II in the *Mecp2* null cortex. We found that loss of MeCP2 affected the binding of RNA pol II in two distinct manners. In cluster I, consisting of primarily paused genes with minimal expression, the binding of RNA pol II was depleted only on the TSS. Conversely, in clusters II and III, consisting of actively transcribing genes, the binding of RNA pol II was depleted both on the TSS and the gene body. These observations prompt us to hypothesize that MeCP2 regulates transcription at both the initiation and elongation stages by recruiting different protein partners. While transcriptional initiation factors that functionally interact with MeCP2 have yet to be identified, we propose that MeCP2 facilitates transcriptional elongation by recruiting the SEC .

While our study reveals a previously unknown mechanism by which MeCP2 regulates gene expression, it also highlights the crucial role of the SEC for proper neurological function. Besides our study, previous work has also highlighted the importance of the SEC in the brain. For example, in *Drosophila*, the SEC is critical for driving neural stem cell fate commitment during brain development (*42*). Furthermore, in humans, various pathogenic mutations in the *AFF* paralogs have been identified to cause multiple neurodevelopmental disorders (*43*–*49*). Despite the importance of the SEC in the brain, not much has been studied about its function and how its dysfunction can lead to neuropathological conditions. Future studies will need to carefully examine the role of the SEC in the brain during both development and adulthood.

In summary, through a genetic modifier screen, we uncovered previously unknown genetic interactors of *MECP2*. These findings reveal that the molecular function of MeCP2 is truly complex, as it can interact with various proteins with diverse functions. This complexity, in turn, suggests that MeCP2 loss of function might disrupt interactions with multiple partners, which may all contribute to the pathogenesis of RTT. By establishing a network of *MECP2* genetic interactors, we have laid the groundwork to further dissect the molecular function of MeCP2, which will allow us to gain a deeper understanding of the pathogenic mechanisms underlying RTT.

## MATERIALS AND METHODS

### *Drosophila* genetic interactor screen

From a collection of potentially druggable human genes related to chromatin biology (538 genes), we identified the *Drosophila* orthologs using the Blast algorithm applied to protein sequences. Using a cutoff *e* value of E-10 yielded 373 *Drosophila* orthologous genes. We then proceeded to screen those genes for which RNAi lines were available (219 genes). We used transgenic UAS-h*MECP2* (second and third chromosome) to construct GMR-Gal4, UAS-h*MECP2*/Cyo; UAS-h*MECP2*/TM6B flies. RNAi alleles were purchased from the Vienna *Drosophila* RNAi Center and the Bloomington Drosophila Stock Center repositories. Flies were crossed at 28.5°C, and the external eye phenotype was imaged with a light microscope. To quantify the severity of the rough-eye phenotype, we measured the eye area showing fused or missing facets using ImageJ and then normalized against the total eye area. Three rounds of quantification were carried out for each genotype (technical replicates) to account for potential sampling error. This gave us the average ratio of disorganized eye area/total eye area for each genotype.

### Animals

All mice used in this study were maintained in a 12-hour light:dark cycle at 20° to 22°C and 30° to 70% humidity, with standard chow and water ad libitum. *Mecp2*^-/y^ and *Mecp2*^fl/y^ mice, described previously (*33*, *50*), were maintained on the 129S6/SvEvTac background. Constitutive *Aff4*^KO/+^ mice (MMRRC, 041408-UCD) were maintained on the 129S6/SvEvTac background. *Aff4* flox mice, described previously (*51*), were maintained on the C57BL/6J background and the *Camk2a*-Cre mouse was purchased from the Jackson Laboratory (JAX, 5359). Only male mice were used for this study to clearly assess the effect of MeCP2 loss, as *Mecp2* is an X-linked gene and female mice are mosaic. For KA injection, KA (Tocris, 0222) was dissolved in 0.9% sodium chloride solution (Henry Schein, 1047098) and 25 mg/kg was injected intraperitoneally. The Baylor College of Medicine Institutional Animal Care and Use Committee approved all research and animal care procedures.

### Cell culture

HEK293T cells were cultured with Dulbecco’s Modified Eagle’s Medium (DMEM; VWR, 10-013-CV) supplemented with 10% heat-inactivated fetal bovine serum and antibiotic-antimycotic (Thermo Fisher Scientific, 15240112). Cells were maintained at 37°C and 5% CO_2_.

### Bimolecular fluorescence complementation

Human *MECP2* (E2 isoform) cDNA was cloned into the pBiFC vector that tags the C-terminal half of YFP on the C terminus. Human *AFF4*, *ENL*, and *EAF1* cDNAs were cloned into the pBiFC vector that tags the N-terminal half of YFP on the C terminus. pBiFC constructs (0.5 μg) expressing *MECP2* and SEC subunits were transfected into HEK293T cells using Lipofectamine 3000 (Thermo Fisher Scientific, L3000150) according to the manufacturer’s instruction. After 48 hours, cells were harvested for either FACS or confocal imaging.

Cells were washed once with phosphate-buffered saline (PBS) and then incubated with the Helix NP NIR dye (BioLegend; 425301), a nucleic acid stain used to discriminate live and dead cells. Cells were acquired and analyzed using a BD LSRFortessa flow cytometer (BD Biosciences). Unstained controls were included to set the baseline fluorescence and to determine background signal. A positive control sample expressing MeCP2-GFP was used to set the positive gate, and a negative control sample expressing MeCP2-cYFP together with empty-nYFP was used to set the negative gate. FACSDiva software was used to collect the data, while FlowJo (v10.8) was used to perform analyses and generate relevant plots.

For confocal imaging, cells were washed once with PBS and then fixed with 4% formaldehyde. After fixation, the cells were permeabilized with PBS containing 0.5% Triton X-100 and then stained with 4′,6-diamidino-2-phenylindole (Thermo Fisher Scientific, 62248). Coverslips with cells were mounted on slides with mounting medium (Thermo Fisher Scientific, P36961), and images were taken with a confocal microscope (Zeiss LSM710).

### Coimmunoprecipitation

For HEK293T cells, a confluent 15-cm plate was lysed in 1 ml of IP buffer [10 mM Hepes (pH 7.9), 3 mM MgCl_2_, 5 mM KCl, 140 mM NaCl, 0.1 mM EDTA, and 0.5% NP-40] that was supplemented with protease and phosphatase inhibitors (Gendepot; P3100-020 and P3200-020, respectively) and 1:1,000 Pierce Universal Nuclease (Thermo Fisher Scientific, 88702). For cortical tissues, a single hemisphere was dounced in 1 ml of IP buffer supplemented with protease and phosphatase inhibitors and nuclease. Lysates from cells and tissues were rotated on an end-over-end rotator for 1 hour at 4°C and then centrifuged at 16,000 rcf at 4°C for 20 min. The supernatant was transferred to a new tube, and input was retrieved before mixing with Dynabead G (Thermo Fisher Scientific, 10004D, 20 μl per IP for cells and 30 μl per IP for tissues) that were preconjugated with one of the following: 5 μg of MeCP2 antibody (Sigma-Aldrich, M6818), 5 μg AFF4 antibody (Proteintech, 14662-1-AP), and 5 μg of normal mouse or rabbit immunoglobulin G (Millipore, 12-371 and 12-370). The supernatant/Dynabead-antibody mixture was rotated overnight at 4°C. The following day, the beads were washed four times with 1 ml of cold IP buffer and subsequently eluted in 25 μl of 1.1× LDS sample buffer/reducing agent (Thermo Fisher Scientific, NP007 and NP009, respectively) at 95°C for 5 min with shaking.

### In vitro binding assay

Full-length MECP2 and its fragment cDNAs were cloned into the pT7CFE1 vector that tags FLAG on the C terminus. SEC component cDNAs were cloned into the pT7CFE1 vector that tags HA on the N terminus. The constructs were used for in vitro transcription and translation using the 1-Step Human High-Yield Mini IVT Kit (Thermo Fisher Scientific, 88891) according to the manufacturer’s instruction. Proteins to be tested for binding were incubated together in 250 μl of IP buffer and incubated for 1 hour at 4°C on an end-over-end rotator. Before IP, 15 μl were taken for input. Ten microliters of the FLAG-bead slurry (Sigma Aldrich, M8823-1ML) was reconstituted in 50 μl of IP buffer per sample and added to the IP samples. After 1 hour of rotation at 4°C, the beads were washed four times with IP buffer. The beads were eluted with 25 μl of 1.1× LDS sample buffer/reducing agent at 95°C for 5 min with shaking.

### Western blot

Cell and tissue lysate samples were run on 4 to 12% bis-tris midi-gels (Invitrogen, WG1403BOX) and transferred to nitrocellulose membranes using wet transfer. Membranes were blocked for 30 min using the INTERCEPT (TBS) blocking buffer (Li-Cor, 927-60010) and then incubated with the following primary antibodies overnight at 4°C: AFF4 (Bethyl Laboratories, A302-538A at 1:1000; Proteintech, 14662-1-AP at 1:5000; and Boster Bio, A03824 at 1:1000), AF9 (Genetex, GTX 102835 at 1:1000), ENL (Cell Signaling Technology; 14893 at 1:1000), ELL2 (Bethyl; A302-505A at 1:1000), total RNA pol II (Cell Signaling Technology; 14958S at 1:1000), pSer^2^ RNA pol II (Millipore, 04-1571 at 1:1000), MeCP2 (Cell Signaling Technology; 3456S at 1:1000), GFP (Abcam; ab13970 at 1:10,000), β-actin (Cell Signaling Technology; 8457S at 1:10,000), vinculin (Sigma-Aldrich; V9131-.2ML at 1:20,000), FLAG (Sigma-Aldrich; F3165-1MG at 1:4000), and HA (Cell Signaling Technology; 3724S at 1:4000). Membranes were washed three times with TBS-T, incubated with IRDye secondary antibodies (Li-COR, 926-68020, 926-32211, and 926-68028 at 1:10,000) and Alexa Flour 790–AffiniPure antibody (Jackson ImmunoResearch; 115-655-174 at 1:10,000) for 1 hour, and then washed three times with TBS-T. Blots were imaged on the Odyssey CLx (LiCoR).

### ChIP sequencing

*Mecp2*^−/y^ (129S6/SvEvTac) female mice were bred to WT (FVB) males, and the cortex was harvested at 7 weeks of age. Tissues were immediately snap-frozen in liquid nitrogen and stored at −80°C until use. One hemisphere of the cortex was used for each ChIP. Tissues were cross-linked with 1% formaldehyde in PBS for 10 min at room temperature and were quenched with 0.2 M glycine for 5 min. Chromatin was sonicated with the Covaris E220 using the following conditions: 6 min, 5% duty cycle, 140 peak intensity power, and 200 cycles per burst for AFF4 and pSer^2^ RNA pol II and 6 min, 10% duty cycle, 140 peak intensity power, and 200 cycles per burst for RNA pol II. The following amounts of *Drosophila* spike-in chromatin (Active Motif, 53083) were added per ChIP: 35 ng for AFF4 and pSer^2^ RNA pol II and 180 ng for RNA pol II. Two micrograms of spike-in antibody (Active Motif, 61686) was added to each sample with one of the following antibodies for IPs: 5 μg of AFF4 antibody (Bosterbio, M03824), 5 μl of RNA pol II antibody (Cell Signaling Technology; 14958S), and 5 μg of pSer^2^ RNA pol II antibody (EMD Millipore; 04-1571). IPs were performed overnight with Dynabeads Protein G. Reverse cross-linking and protein degradation were performed at 65°C overnight. IPed DNA was purified using the QIAquick polymerase chain reaction (PCR) purification kit (Qiagen, 28106). DNA libraries were prepared using the KAPA Hyperprep kit (Roche; KK8504). The cycle number for library amplification was empirically determined using SYBR Green I Nucleic Acid Gel Stain (Thermo Fisher Scientific, S7585) and real-time quantitative PCR tracking (Bio-Rad CFX96). Pooled libraries were sequenced on the NovaSeq 6000 (Illumina) using single-end 100 cycle or paired-end 50 cycle reads.

### Total RNA-seq

*Mecp2*^+/−^ (129S6/SvEvTac) female mice were bred to WT (FVB) males, and the cortex was harvested at 7 weeks of age. Tissues were immediately snap-frozen in liquid nitrogen and stored at −80°C until use. One cortical hemisphere was used to extract RNA using the RNeasy kit (Qiagen, 74106). Briefly, tissues were homogenized in buffer RLT using a tissue homogenizer (Cole Parmer). Homogenate was passed through a QIAshredder (Qiagen, 79656), and the flow through was processed with the RNeasy kit (Qiagen, 74104). Ribosomal RNA was depleted using the NEBNext rRNA Depletion Kit v2 (NEB, E7400L), and libraries were prepared using the NEBNext Ultra II Directional RNA Library Prep Kit for Illumina (NEB, E7770L). Pooled libraries were sequenced on the NovaSeq 6000 instrument using paired-end 51 cycle reads.

### Bioinformatic analysis

For ChIP-seq, reads were trimmed using Cutadapt v4.1 with parameters --nextseq-trim = 30 --minimum-length = 20 and then aligned to the mm10 mouse genome assembly and dm6 *Drosophila* genome assembly for spike-in read mapping using Bowtie2 v2.2.6 (*52*) with parameters --sensitive --no-unal. Peaks were called using MACS2 (*53*) with parameters -q 0.05 --keep-dup auto --nomodel, and then annotated using HOMER v4.1.1. Reads from mouse fragments were normalized to the *Drosophila* spike-in reads. Protein coding gene detection and selection, TSS and gene-body region selection, and coverage mapping were performed using in-house R and perl scripts. We filtered genes based on the following criteria: (i) RNA pol II occupied genes, (ii) longer than 2 kilobases (kb), and (iii) neighboring genes that are at least 2 kb apart. This resulted in a total of 8696 genes that were used for downstream analysis. Occupancy and log_2_ fold change heatmaps were generated using deepTools v.3.5.1 (*54*). For the hierarchical clustering analysis, we clustered on the basis of two of three replicates in which the *Mecp2* null samples showed the greatest difference from WT samples. For correlation plots, ChIP-seq gene-level data were summarized as median of ratios of *Mecp2* null to WT occupancy, across replicates. This approach does not compress replicate-level information and reflects consistency of the impact of *Mecp2* loss in matched animal pairs.

For total RNA-seq, the output data were processed with bcl2fastq. Sequence quality was assessed using FastQC v 0.11.2, and quality trimming was done using Trimmomatic (*55*). RNA-seq reads were aligned to the mm10 genome using STAR v.2.5.2 (*56*), and only uniquely mapped reads with a two-mismatch threshold were considered for downstream analysis and then quantified to the gene level using HTSeq (*57*). Output bam files were converted into bigwig track files to display coverage throughout the genome (in RPM) using the GenomicRanges package (*58*) as well as other standard Bioconductor R packages. Gene count tables were constructed following Ensembl gene annotations and used as input for Remove Unwanted Variation from RNA-Seq Data (RUVseq) normalization (*59*) and differential gene expression analysis was performed using DESeq2 (*60*). Genes with adjusted *P* values (Benjamini-Hochberg) of less than 0.05 were treated as differentially expressed.

### Behavioral test

*Mecp2*^fl/+^ (129S6/SvEvTac) female mice were bred to *Aff4*^KO/+^ (129S6/SvEvTac) male mice, and the resulting male progenies were aged until 10 weeks of age. Mice were tested on the open-field, rotarod, and fear conditioning by an individual who was blinded to the genotypes. Mice were given at least 1 day to recover between different behavioral assays.

#### Open-field assay

The illuminance in the testing room was set to 150 lux, and the background noise level was set to 62 dB using a white noise generator. After 30 min of habituation in the testing room, mice were placed in a 40 cm by 40 cm by 30 cm arena equipped with photobeams. The mice were placed in the arena for 30 min, and their movement was recorded and analyzed using the AccuScan Fusion software.

#### Rotarod

The illuminance in the test room was set to 150 lux, and the background noise level was set 62 dB using a white noise generator. After 30 min of habituation, the mice were placed on an accelerating rotarod apparatus (Ugo Basile). The rotarod accelerated from 4 to 40 rpm, and the mice were tested for a maximum of 10 min. Mice were tested four trials per day for four consecutive days. The time it took for mice to fall from the rotarod was recorded manually.

#### Fear conditioning

Mice were habituated in a room adjacent to the testing room that had illuminance level of 150 lux and background noise level of 62 dB. The day before testing, the mice were brought to the testing room and placed inside a chamber containing a grid floor that can deliver an electric shock. The mice were trained using a paradigm consisted of 2 min of silence and then a tone for 30 s (5 kHz and 80 dB), which is followed immediately by a foot shock that lasts for 2 s (1 mA). The tone and foot shock were repeated after 2 min of silence. The mice were returned to their home cages after training. Twenty-four hours later, context and cued tests are performed. During the context test, the mice were placed in the same chamber with no noise or any foot shock for 5 min. After 1 hour, cued test was performed where the mice were placed in a novel environment for a total of 6 min. For the first 3 min, the mice were left in silence, and then for the next 3 min, a tone (5 kHz and 80 dB) was given. The movement of mice were recorded on video, and freezing episodes were quantified using the FreezeFrame software (ActiMetrics).

### Statistical analysis

One-way analysis of variance (ANOVA) followed by Dunnett’s multiple comparison test was used to compare protein levels across different genotypes in *Drosophila* and mice. Wilcoxon rank sum test was used to compare the average of RNA-seq gene counts across clusters between WT and *Mecp2* null mice. One-way ANOVA followed by Holm-Sidak’s multiple comparison test was used to analyze body weight, open field, and fear conditioning behavior. Two-way ANOVA with repeated measures followed by Holm-Sidak’s multiple comparison test was used to analyze rotarod behavior.

## Supplementary Materials

### The PDF file includes:

Figs. S1 to S6 Full blot from which Fig. 2C originated from Legends for tables S1 to S3
