# Supplementary material for: MeCP2 interacts with the super elongation complex to regulate transcription
Source: Sci Adv. 2025 Nov 26;11(48):eadt5937. doi: 10.1126/sciadv.adt5937 (PMC12652325; doi:10.1126/sciadv.adt5937)
Supplement: Supplementary file 1 — Figs. S1 to S6 Full blot from which Fig. 2C originated from Legends for tables S1 to S3 [file sciadv.adt5937_sm.pdf]

Science Advances
AAAS

Supplementary Materials for

MeCP2 interacts with the super elongation complex to regulate transcription

Jun Young Sonn *et al.*

Corresponding author: Ali Shilatifard, ash@northwestern.edu; Huda Y. Zoghbi, hzoghbi@bcm.edu

Sci. Adv. **11**, eadt5937 (2025)
DOI: 10.1126/sciadv.adt5937

The PDF file includes:

Figs. S1 to S6
Full blot from which Fig. 2C originated from
Legends for tables S1 to S3

Other Supplementary Material for this manuscript includes the following:

Tables S1 to S3

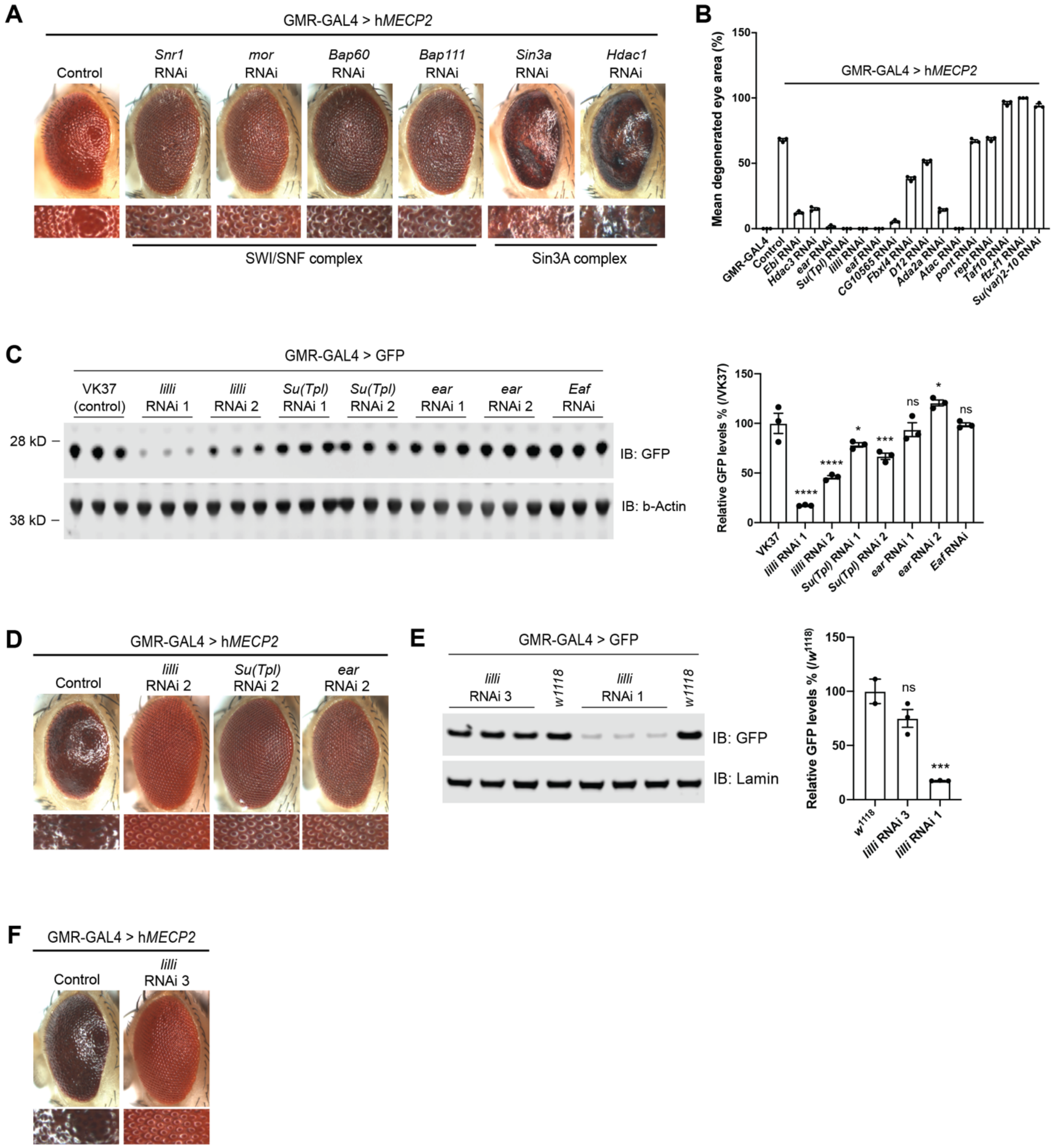

Fig. S1. Knockdown of previously identified MeCP2 interactors in *Drosophila* modify the h*MECP2*-induced rough eye phenotype. (A) RNAi-mediated knockdown of components of the SWI/SNF and Sin3A complexes modify the h*MECP2*-induced rough eye phenotype. (B) Quantification of rough eye phenotypes in Fig. 1C. n = 3 technical replicates. (C) Western blot image and quantification showing the effect of SEC subunit knockdown on GMR-driven GFP expression levels in fly heads. n = 3 biological replicates were used for each genotype. One-way

ANOVA was used for statistical comparison. ns = not significant, * $p<0.05$, *** $p<0.001$, **** $p<0.0001$. (D) Knockdown of SEC components using a second RNAi line suppresses the h*MECP2*-induced rough eye phenotype. (E) Western blot image and quantification showing that a third RNAi line against *lilli* reduces GFP levels by 25%. 2-3 biological replicates were used for each genotype. One-way ANOVA was used for statistical comparison. (F) Knockdown of *lilli* using a third RNAi line suppresses the h*MECP2*-induced rough eye phenotype.

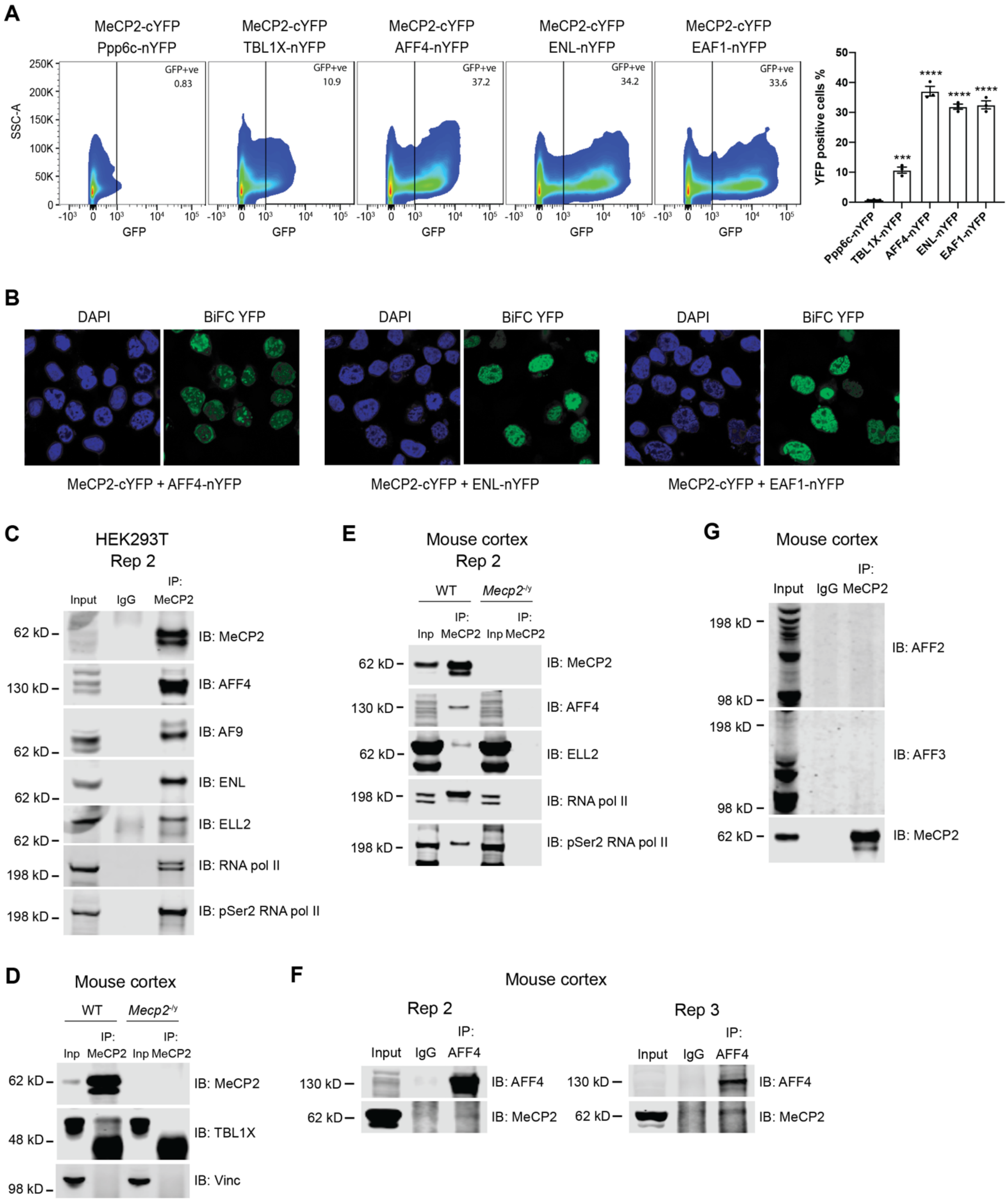

Figure S2. MeCP2 interacts specifically with AFF4-associated SEC. (A) FACS analysis shows that co-transfection of MeCP2-cYFP and Ppp6c-nYFP does not generate YFP-positive

cells, whereas co-transfection with TBL1X-nYFP yields YFP-positive cells. Similarly, co-transfection of MeCP2-cYFP and nYFP-tagged SEC subunits (AFF4, ENL, and EAF1) yielded YFP-positive cells. Quantification shows the proportion of YFP-positive cells. n = 3 independent experiments. *** $p<0.001$, **** $p<0.0001$. (B) Confocal images showing BiFC YFP signals inside the nucleus when MeCP2-cYFP is co-transfected with nYFP-tagged SEC subunits. (C) Independent experiment of MeCP2 IP in HEK293T cells shows co-IP of SEC subunits and RNA pol II. (D) MeCP2 IP from WT mouse cortical lysate shows co-IP of TBL1X (positive control), but not Vinculin (negative control). (E) Independent experiment of MeCP2 IP in the mouse cortex shows co-IP of AFF4, ELL2, and RNA pol II. (F) Independent experiments of AFF4 reverse IP from WT mice cortical lysates show co-IP of MeCP2. Normal rabbit IgG was used as a negative control. Different brightness settings were used for the top and bottom blots due to the relatively weaker MeCP2 co-IP band intensity compared to the AFF4 IP band intensity. Immunoblotting against AFF4 for (C) and (E) were done with the Bethyl laboratories antibody (A302-538A), whereas IP and immunoblotting against AFF4 for (F) was done with the Proteintech antibody (14662-1-AP). (G) Western blot images showing the absence of co-IP between MeCP2 and other AFF paralogs (AFF2 and AFF3).

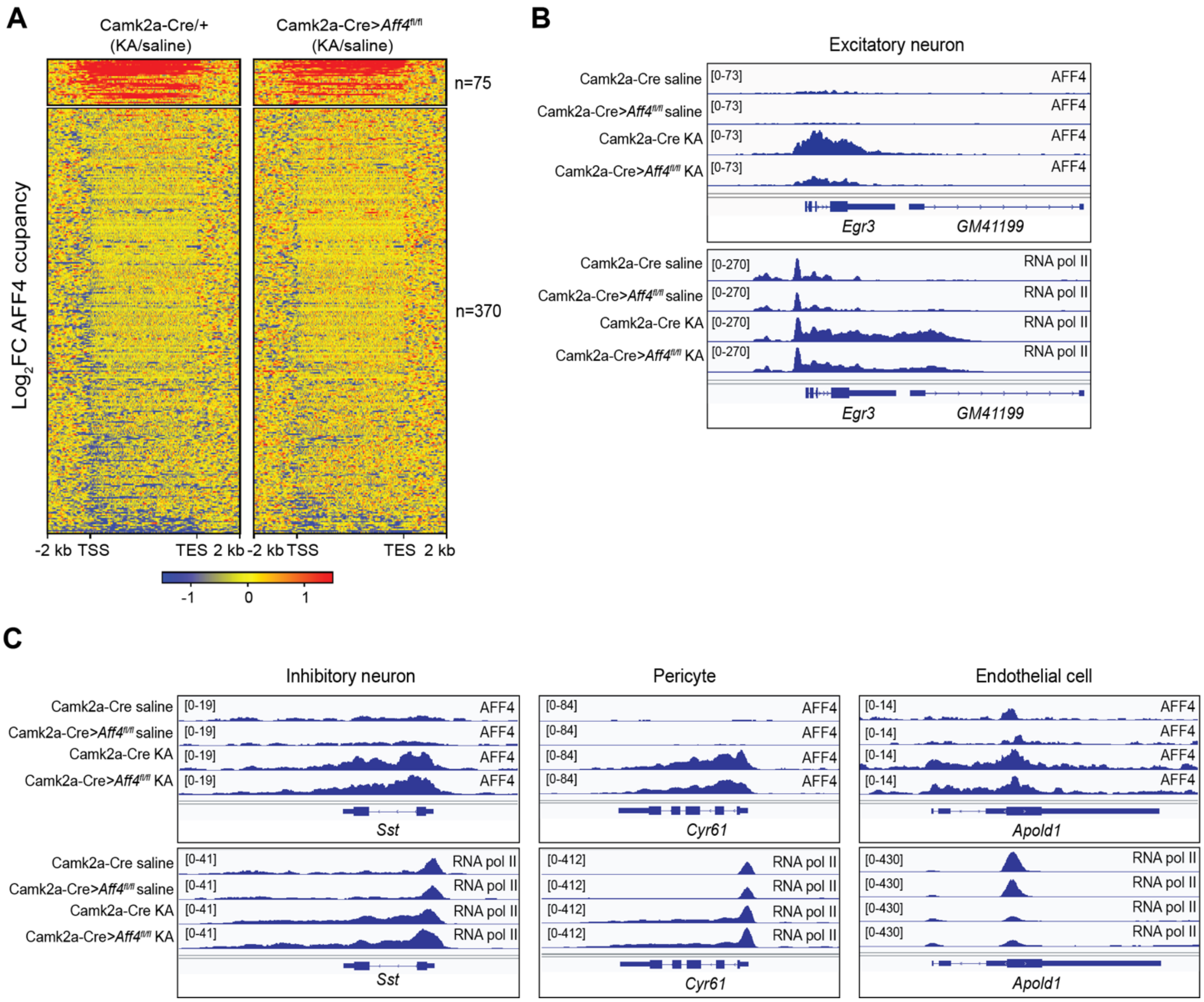

Figure S3. AFF4 antibody validation for ChIP-seq in the mouse brain. (A) Global heatmap showing the log2 fold change of AFF4 occupancy after KA injection (45 mins) compared to baseline (saline injection) in the cortex of control (Camk2a-Cre/+) and *Aff4* cKO (Camk2a-Cre>*Aff4*$^{fl/fl}$) mice. (B) Track example showing that KA-induced binding of AFF4 and release of RNA pol II on *Egr3* are suppressed by depletion of AFF4 in excitatory neurons. (C) Track examples showing that KA-induced binding of AFF4 is not suppressed on genes that are expressed outside of excitatory neurons.

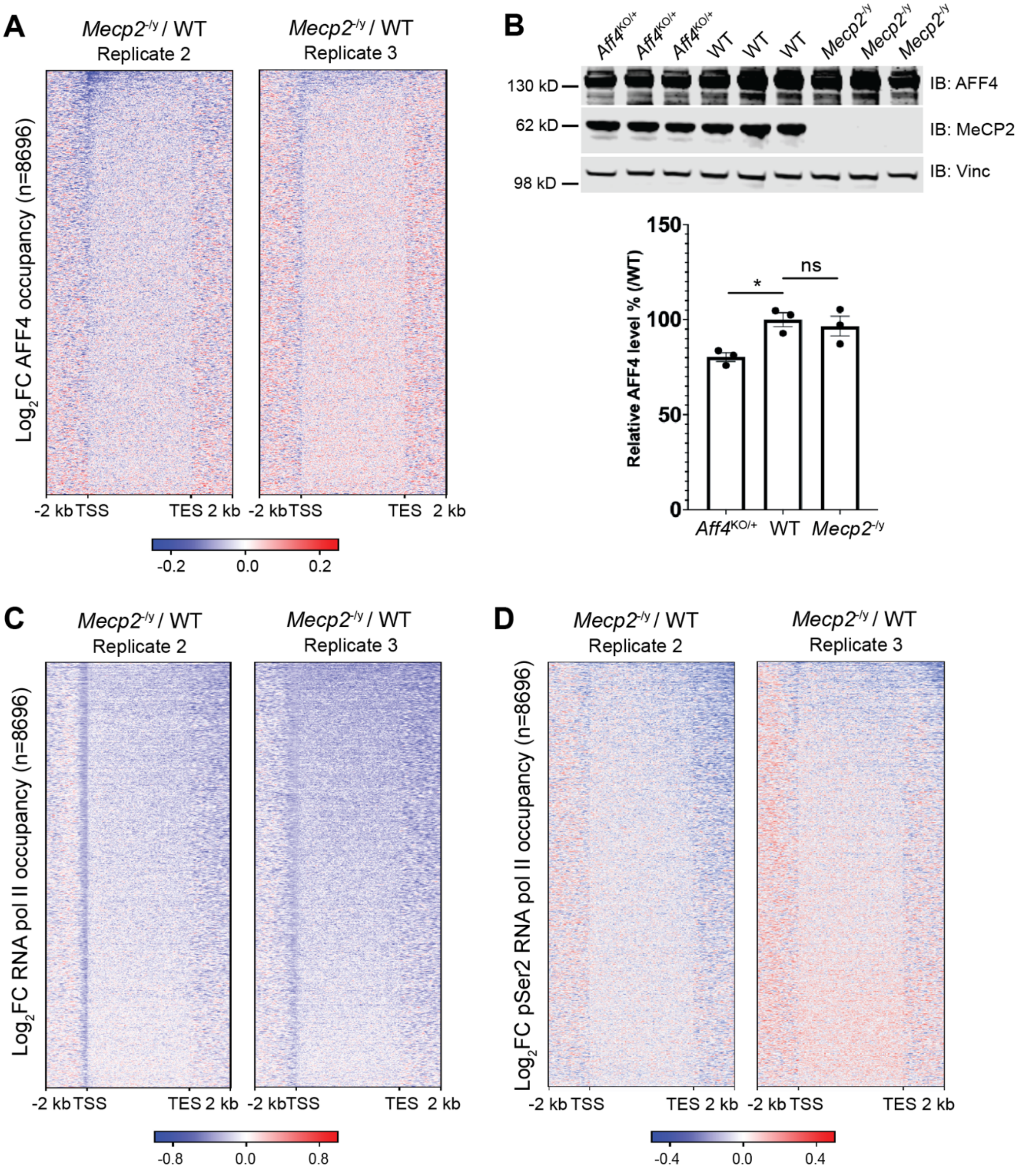

Figure S4. AFF4, RNA pol II, and pSer2 RNA pol II ChIP-seq in other biological replicates. (A) Heatmaps of log2 fold change of AFF4 occupancy in *Mecp2* null mice compared to WT mice in two other biological replicates. (B) Western blot image and quantification showing that loss of MeCP2 has no effect on AFF4 protein levels in the mouse cortex. An AFF4 antibody from Boster Bio (A03824) was used for Western blot. n = 3 biological replicates were used for each genotype. ns = not significant and * p<0.05. (C) Heatmaps of log2 fold change of

RNA pol II occupancy in *Mecp2* null mice compared to WT mice in two other biological replicates. (D) Heatmaps of log2 fold change of pSer2 RNA pol II occupancy in *Mecp2* null mice compared to WT mice in two other biological replicates.

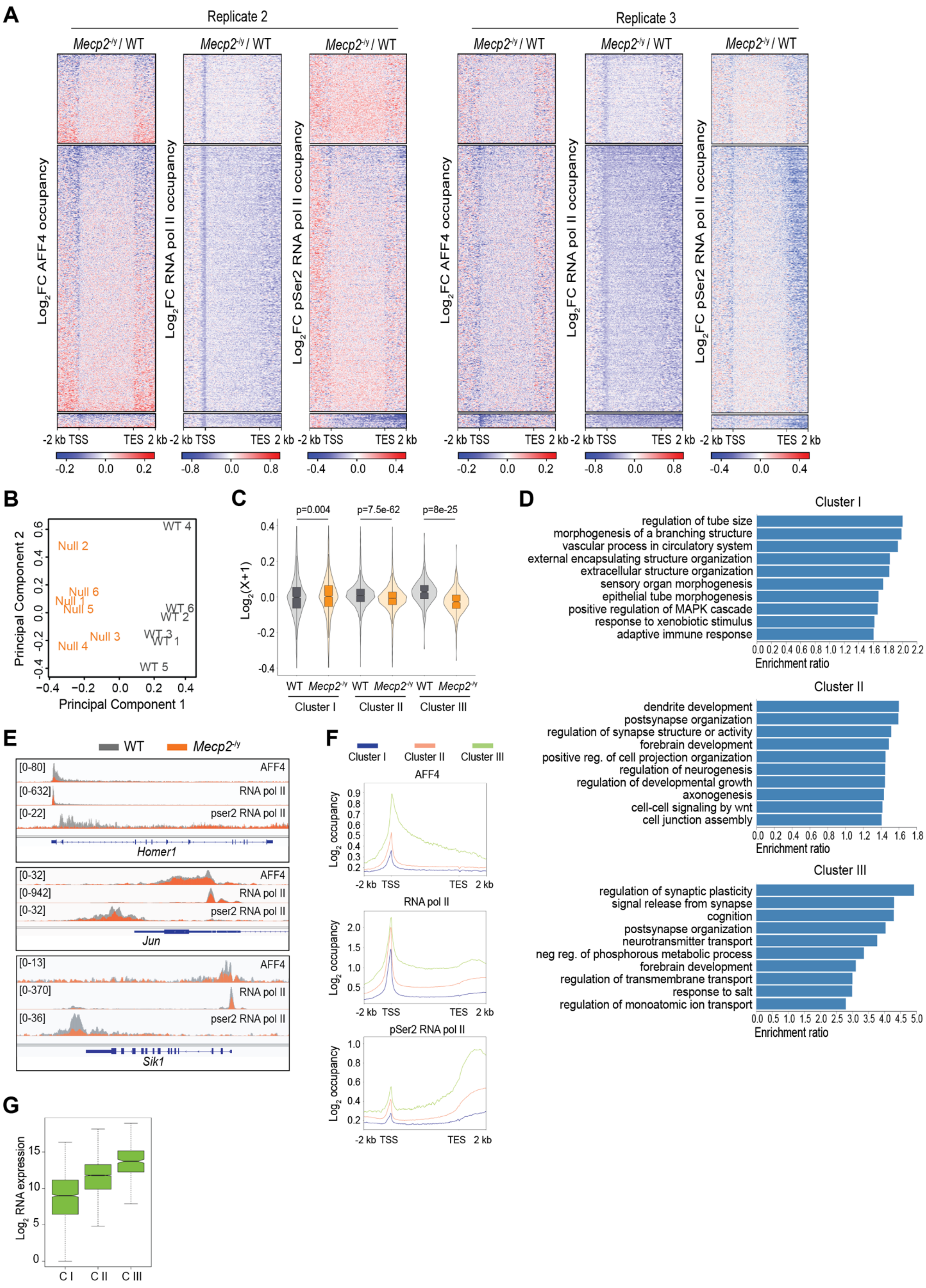
A
Replicate 2
Replicate 3
Mecp2-/y / WT
Log2FC AFF4 occupancy
Log2FC RNA pol II occupancy
Log2FC pSer2 RNA pol II occupancy
-2 kb TSS
TES 2 kb
B
Principal Component 1
Principal Component 2
C
p=0.004
p=7.5e-62
p=8e-25
Log2(X+1)
Cluster I
Cluster II
Cluster III
D
Cluster I
regulation of tube size
morphogenesis of a branching structure
vascular process in circulatory system
external encapsulating structure organization
extracellular structure organization
sensory organ morphogenesis
epithelial tube morphogenesis
positive regulation of MAPK cascade
response to xenobiotic stimulus
adaptive immune response
Enrichment ratio
Cluster II
dendrite development
postsynapse organization
regulation of synapse structure or activity
forebrain development
positive reg. of cell projection organization
regulation of neurogenesis
regulation of developmental growth
axonogenesis
cell-cell signaling by wnt
cell junction assembly
Enrichment ratio
Cluster III
regulation of synaptic plasticity
signal release from synapse
cognition
postsynapse organization
neurotransmitter transport
neg reg. of phosphorous metabolic process
forebrain development
regulation of transmembrane transport
response to salt
regulation of monoatomic ion transport
Enrichment ratio
E
WT
Mecp2-/y
AFF4
RNA pol II
pser2 RNA pol II
Homer1
Jun
Sik1
F
Cluster I
Cluster II
Cluster III
AFF4
RNA pol II
pSer2 RNA pol II
Log2 occupancy
G
Log2 RNA expression
C I
C II
C III

Figure S5. AFF4, RNA pol II, and pSer2 RNA pol II ChIP-seq clustering other biological replicates and total RNA-seq. (A) Clustered heatmaps showing the log2 fold change of AFF4, RNA pol II, and pSer2 RNA pol II occupancy in *Mecp2* null mice compared to WT mice in other biological replicates. (B) Principal component analysis plot of RNA-seq samples. (C) Violin plot showing the log2-transformed average expression of genes in each cluster relative to WT cluster I. X stands for replicate-averaged RNA-seq counts. $n = 6$ biological replicates. (D) Gene ontology analysis of clusters I-III. All terms are FDR < 0.05. (E) Track examples of AFF4, RNA pol II, and pSer2 RNA pol II binding on three different activity-dependent genes. (F) Profile plots of averaged occupancy over the gene and 2 kb flanking regions of AFF4, RNA pol II, and pSer2 RNA pol II in a representative WT mouse across each cluster. (G) Boxplot of replicate-averaged log2-transformed RNA-seq counts in each cluster in WT mice. C1 = cluster I, C2 = cluster II, and C3 = cluster III.

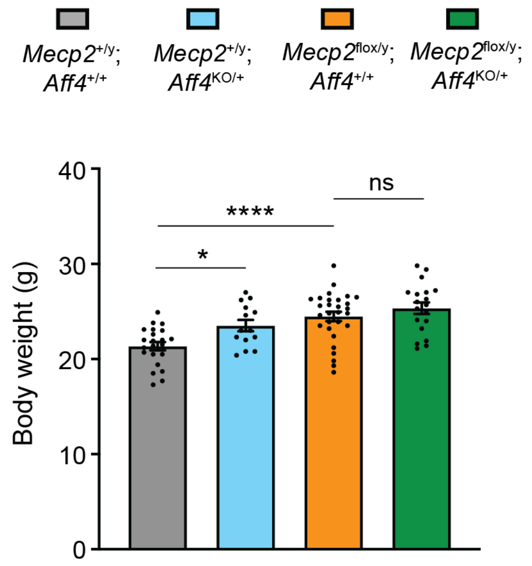

Figure S6. Body weight of WT, *Mecp2*^{flox}, *Aff4*$^{KO/+}$, and double mutant mice. Measurements at 12-weeks of age reveal that *Mecp2* and *Aff4* do not genetically interact to regulate body weight. n = 22 wildtype mice; n = 14 *Aff4* heterozygous knockout mice; n = 29 *Mecp2* hypomorphic mice; n = 19 double mutant mice. ns = not significant, * $p<0.05$ and **** $p<0.0001$.

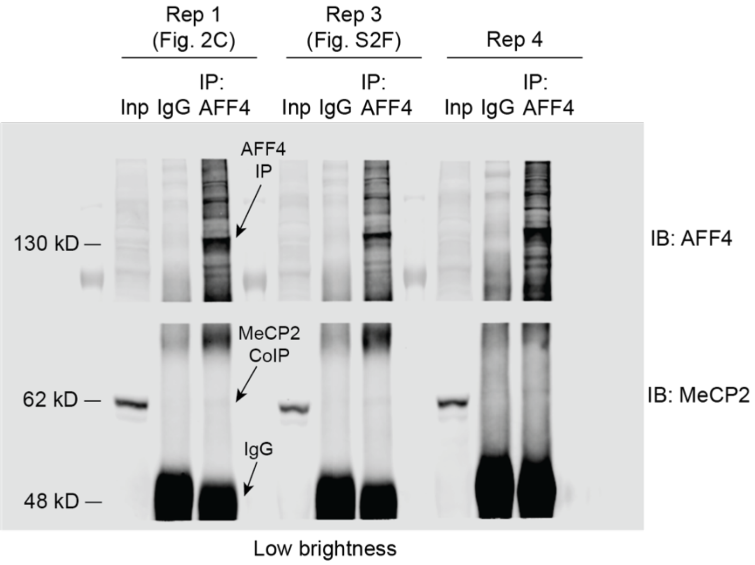

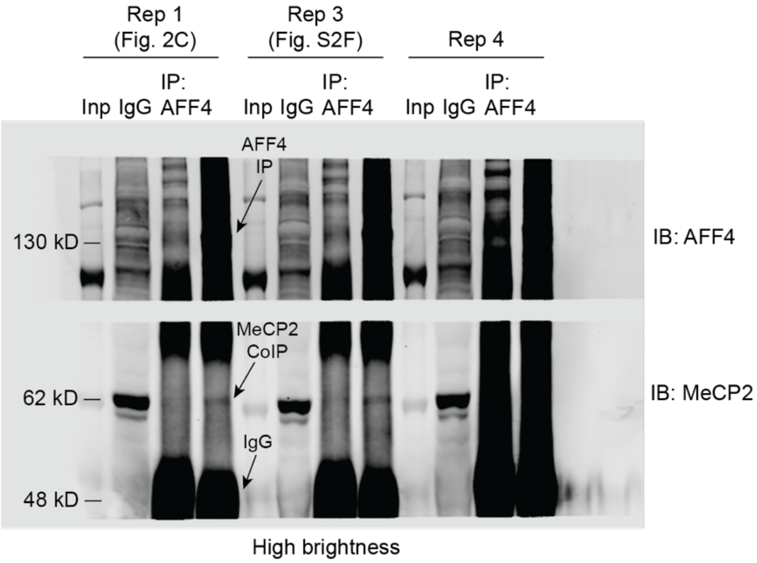

Full blot from which Fig. 2C originated from. Blot using low brightness (top blot) shows IP of AFF4 from cortical lysates of three independent WT mice (Rep1, Rep 3, and Rep 4; Rep 2 included in the manuscript was performed in an independent experiment). At the same brightness, the co-IP band for MeCP2 is extremely faint. While the co-IP band for MeCP2 becomes more discernible using higher brightness (bottom blot), this causes an oversaturation of the AFF4 IP band. For this reason, we applied different brightnesses for the AFF4 and MeCP2 immunoblots. Inp = input; IgG = control IP using normal IgG.

Table S1. List of Drosophila genes used for the genetic modifier screen. List of 219 chromatin-associated *Drosophila* genes that were screened in this study.

Table S2. Genes showing altered AFF4 binding in the mouse cortex upon KA injection. Genes that exhibit altered AFF4 binding in *Camk2a*-Cre/+ mouse cortex upon KA injection (45 minutes). Cluster 1 represents genes with increased binding (75 genes) and cluster 2 represents genes with decreased binding.

Table S3. Gene list for clusters I-III. List of genes clustered based on AFF4 and RNA pol II binding change in *Mecp2* null mice compared to WT mice.
